# Tumor-driven stromal reprogramming in the pre-metastatic lymph node

**DOI:** 10.12688/f1000research.145171.1

**Published:** 2024-03-27

**Authors:** Michelle Piquet, David A Ruddy, Viviana Cremasco, Jonathan Chang

**Affiliations:** 1 Oncology Innovative Targets and Technologies, Novartis, Cambridge, MA, 02139, USA; 2 Oncology Translational Research, Novartis, Cambridge, MA, 02139, USA

**Keywords:** Tumor Draining Lymph Node, scRNAseq, metastasis, Stromal Cell, Fibroblast, Endothelial Cell, pre-metastatic Niche

## Abstract

**Background:**

Metastatic dissemination is critically reliant on the formation of a receptive niche, a process which is thought to rely on signals derived from the primary tumor. Lymph nodes are continuously exposed to such signals through the flow of afferent lymph, allowing the potential reprograming of lymphoid tissue stroma in support of metastases or immunosuppression. The objective of this study was therefore to better characterize tumor-driven transcriptomic changes occurring to specific stromal populations within the tumor-draining lymph node.

**Methods:**

We utilize single cell RNA sequencing of dissociated LN tissue extracted from tumor-bearing and naïve mice to profile the reprograming of tissue stroma within the pre-metastatic lymph node.

**Results:**

Resulting data provides transcriptomic evidence of tumor-induced imprinting on marginal reticular cells (MRCs) and floor lymphatic endothelial cells (fLECs) populating the subcapsular sinus. These alterations appear to be unique to the tumor-draining LN and are not observed during inflammatory antigenic challenge. Notably, MRCs exhibit characteristics reminiscent of early desmoplastic CAF differentiation, fLECs engage distinct chemoattractant pathways thought to facilitate recruitment of circulating cancer cells, and both stromal populations exhibit signs of metabolic reprograming and immune-modulating potential.

**Conclusions:**

Cumulatively, these findings build upon existing literature describing pre-metastatic niche formation and offer several promising avenues for future exploration.

## Introduction

Accumulation of genetic changes underpinning malignant transformation is the trigger point of cancer initiation. ^1^^,^^2^ However, these cell intrinsic alterations alone are insufficient for successful growth and progression of solid tumors. Rather, survival of the nascent tumor requires a co-evolved and permissive microenvironmental niche, wherein tissue stroma must be coopted to provide the structural, metabolic, and immune evasive support network necessary for tumor growth and eventual metastatic dissemination. ^1^^–^^3^ The therapeutic potential of exploiting this interdependence has spurred significant effort to unravel the complexities of the tumor microenvironment, though the processes by which normal tissues are rendered tumor-permissive remain incompletely understood.

Specific microenvironmental requirements likewise exist for the formation of secondary tumor growth, as the engraftment and survival of circulating cancer cells is equally reliant on the existence of a receptive niche. Reflective of these exigencies, fewer than 0.01% of circulating tumor cells are thought to successfully establish tumor metastases. ^4^ Moreover, the site of eventual metastatic engraftment is non-random, with specific organotropic patterns varying by cancer indication – an observation which serves as the basis of Stephen Paget’s longstanding “seed and soil” hypothesis. ^5^^–^^7^ Mounting evidence now suggests that successful metastasis additionally follows a reprograming of stromal elements at these distant sites, mediated by secretion of factors and extracellular vesicles originating from the primary tumor. ^8^^–^^13^ The resulting modifications form a more favorable microenvironment, termed the premetastatic niche (PMN). ^9^ Decoding these microenvironmental alterations and identifying the affected cell types may open a window to the requisite conditions for early tumor cell engraftment, growth, and immune evasion, and potentially reveal new points of therapeutic intervention. However, in-depth study of these processes may be experimentally challenging given variability in exact timing and location of metastases.

More predictable is the spread of cancer cells to the regional lymphatic lymph node (LN), which are frequently the first site of tumor metastasis in most forms of carcinoma. ^14^ This prevalence of metastatic spread to the draining LN is almost certainly a factor of direct exposure to tumor-derived signals, as such signals pool within the afferent lymphatics before invariably draining to the sentinel node. ^15^^–^^19^ Moreover, lymphangiogenesis and increased lymph flow is believed to be an essential step that precedes LN metastasis. ^15^^,^^20^^,^^21^ While exposure to afferent lymphatic flow facilitates the requisite acquisition of antigens and tissue signals for functional adaptive immunity, drainage of tumor-derived factors has the potential to modulate these responses, thereby conditioning the pre-metastatic LN in favor of tumor support and immune suppression. ^22^ Indeed, recent reports have demonstrated tumor-induced alterations to LN fibroblastic reticular cells (FRCs) – a stromal population which comprises the essential infrastructure supporting lymphocyte homeostasis and coordinating immune interactions. ^23^^–^^25^ Changes to FRCs of the tumor draining LN (tdLN) included increased activation and proliferation, disrupted homeostatic chemokine production driving aberrant immune cell localization, and altered matrix production. However, while the observed reprograming of LN stroma has a putative role in metastasis support and immune suppression, many of the described alterations overlap with the stromal response program associated with general inflammation and infection. ^26^ In this regard, the degree to which these previously reported findings are in fact unique or specific to tumor-derived factors requires further examination.

Here we expand on previous exploration of tumor-induced reprogramming through single cell transcriptomic analysis of stroma-enriched samples, enabling a more robust exploration of subset-specific transcriptional shifts. We demonstrated exquisite specificity in stromal reprograming associated with the premetastatic tdLN, wherein select subsets of FRCs and lymphatic endothelial cells (LECs) populating the LN subcapsular sinus (SCS) are differentially affected by tumor-derived signals. Amongst these findings, we confirm functional alterations reflective of matrix remodeling, identify engagement of distinct axes of chemotaxis, and identify transcriptional signs of altered metabolism and immunomodulatory potential. Importantly, these alterations were additionally compared to LN stromal responses following antigenic challenge in the absence of live tumor cells, allowing confirmation of the tumor-specificity of these response programs. Ultimately, we believe these findings help to broaden our understanding of pre-metastatic communication and tissue imprinting, and may direct future experimental exploration of metastatic niche dependencies with potential for therapeutic intervention.

## Methods

### Ethics statement

All animal work was approved and performed in accordance with the guidelines from the Institutional Care and Use Committee (IACUC) at Novartis Institutes for BioMedical Research (Protocol 20 IMO 035) and in compliance with the Guide for the Care and Use of Laboratory Animals. All efforts were made to ameliorate any potential suffering of animals.

### Mice

Experiments were performed in eight-week-old, sex-matched C57Bl/6 mice purchased from Charles River Laboratories. Mice were maintained under specific pathogen-free conditions in accordance with institutional and National Institute of Health guidelines.

### Cell lines

MC38 cells were received from NCI under MTA# 38699-15, expanded, frozen and collected in the NIBR cell line repository. Cell lines were maintained in Dulbecco’s Modified Eagle Medium (Gibco, 11965-092) containing 10% heat-inactivated FBS (VWR, 1500-500), 1% Penicillin-Streptomycin (Gibco, 15140-122), and 1% L-glutamine (Gibco, 25030-81) for one week prior to implant. Prior to inoculation into recipient animals, cell lines were tested and found to be free of mycoplasma and viral contamination in the IMPACT VIII PCR assay panel (IDEXX BioResearch, Missouri).

### Tumor models

The present study used tissues collected from naïve mice (n=4), mice bearing live MC38 tumors (n=3), or fixed MC38 tumor cells (n=3). Mice were randomly assigned to treatment groups. In preparation for inoculation, MC38 cells were detached using 0.25% trypsin, washed, and resuspended in sterile PBS prior to implantation. For the “fixed cell” control treatment condition, MC38 cells were additionally incubated in 4% paraformaldehyde in sterile PBS for 20 minutes at a concentration of 10 ^6^ cells/mL before washing and resuspension in sterile PBS. For both conditions, 5 × 10 ^5^ cells were implanted subcutaneously on the animal’s lower-right flank. Animals are anesthetized by isoflurane inhalation prior to treatment. Mice were monitored for tumor growth, changes in body weight and condition 3 times per week. Mice were euthanized by CO _2_ inhalation on day 12 after treatment, and both the tumor-draining and contralateral non-draining inguinal lymph nodes were collected. No animals were excluded from analysis.

### Enzymatic digestion of lymph nodes

Single-cell suspensions of LNs were prepared for analysis by flow cytometry or single cell RNAseq. LNs were dissected and incubated at 37 °C in RPMI containing 0.1 mg ml ^−1^ DNase I (Invitrogen), 0.2 mg ml ^−1^ collagenase P (Roche) and 0.8 mg ml ^−1^ dispase (Roche) for 50–60 min, as previously described. Liberated cells in suspension were collected into cold medium containing 2% fetal bovine serum and 5 mM EDTA every 15–20 min and replaced with fresh digestion medium. Following complete digestion of the LN, cells were passed through a 70-μm cell strainer, washed and resuspended in cold PBS.

### Flow cytometric analysis

Single-cell suspensions were resuspended in flow cytometry buffer (PBS, 2% FBS, and 2 mmol/L EDTA). Cells were blocked with Fc block and then stained with different fluorochrome-conjugated antibodies for 20 min on ice. The following antibodies were used (clone, fluorophore, concentration): anti-CD45 (30-F11, BUV396, 1:500), anti-CD31 (390, Pacific Blue, 1:300), and anti-PDPN (8.1.1, APC, 1:300). CountBright Absolute Counting Beads (Invitrogen, C36950) were included for quantification of cell counts. Samples were analyzed on a BD flow cytometry Fortessa instrument and analyzed with FlowJo. Raw data available. ^27^

### Stromal enrichment

Single cells obtained form LN digestion were resuspended in mouse FC block (Miltenyi #130-092-575). Biotinylated PDPN (8.1.1, 1:100) antibody was used for positive selection, using the EasySep Selection Kit (Stemcell Technologies #18559). Cells were then washed in cold PBS, counted, and resuspended at 10 ^6^ cells/mL for sequencing.

### Single-cell RNA sequencing and data pre-processing

The 10x Genomics Chromium Single Cell 3’ Reagents v3 kit (10× Genomics, Pleasanton, CA) was used under standard conditions and volumes to process cell suspensions for 3’ transcriptional profiling. The volumes of cell suspensions were calculated to achieve a target cell recovery of 6000 cells for all dissociated samples, following the manufacturer’s guidelines. The resultant purified cDNAs were quantified on an Agilent Tapestation (Agilent, Santa Clara, CA) using High Sensitivity D5000 ScreenTapes and Reagents. The final single cell 3’ libraries were quantified using an Agilent Tapestation using High Sensitivity D1000 ScreenTapes and Reagents. Following this, the libraries were diluted to a concentration of 10 nM in Qiagen Elution Buffer, subjected to denaturation, and loaded onto an Illumina MiSeq at 6 μM, utilizing the MiSeq Reagent Kit v3 (Illumina, San Diego, CA) to assess sample quality and achieve loading normalization for the HiSeq4000. The normalized libraries were loaded onto an Illumina cBOT at a concentration of 160 picomolar and sequenced on a HiSeq4000. The sequencing process included a 28-base-pair first read, followed by two 8-base-pair index reads, and concluded with a 91-base-pair second read, all by using 2 HiSeq4000 SBS kits at 50 cycles each. All sequence intensity files were generated using the Illumina Real Time Analysis software. The resulting intensity files were then subjected to demultiplexing and aligned to the mouse genome, version mm10, using the 10x Genomics CellRanger v3.0.1 software package.

### Single-cell RNA-Seq data quality control and processing

All computational analyses and visualizations were conducted within the R programming environment, using version 3.6.3. The Seurat package (v3.2.3) was employed for quality control and downstream analysis of single-cell RNA-seq data. ^28^ The count matrices were then subjected to comprehensive analysis using the Seurat workflow. Cells that exhibited low quality characteristics, defined as those with fewer than 200 expressed genes, more than 6000 expressed genes, or exceeding 30% mitochondrial content, were excluded from the dataset. Following this, the count matrices were merged and normalized using *SCTransform.* Principal component analysis was executed, focusing on 2000 highly variable genes. To select the appropriate number of principal components (PCs), the Elbow plot heuristic was used. A shared nearest neighbor (SNN) graph was constructed using the first 20 PCs through Seurat’s *FindNeighbors* function, and cell clustering was performed using a Louvain algorithm with *FindClusters.* A resolution parameter of 0.8 was chosen to generate an extensive collection of cell clusters, effectively capturing diverse cell types. Cluster markers were identified using Seurat’s *FindAllMarkers* function. Dimensionality reduction was visualized using the uniform manifold approximation and projection (UMAP) method.

### Clustering and analysis of fLEC, MRC, BRC

The dataset was methodically divided into subsets, specifically isolating floor lymphatic endothelial cells (fLEC), marginal reticular cells (MRC), and b-zone reticular cells (BRC). These subsets underwent a re-clustering process using 15 principal components (PCs) at a resolution parameter of 0.3, with the optimal number of PCs determined using the Elbow heuristic.

### Differential gene expression and pathway enrichment analyses

Differential gene expression analyses were conducted on each subset of cells with an average log-fold change above 0.25, as part of the Seurat workflow. This analysis compared Live Draining vs. Live Non-Draining cells. Pathway enrichment analyses were executed using the *ClusterProfiler* package (v3.14) and its *enrichGO* function, using Biological Process, Cellular Component, and Molecular Function ontologies. ^29^ Pathway enrichment was computed for all cluster marker genes within fLEC, MRC, and BRC subsets, specifically focusing on those with an avg_logFC > 0.25 and an adjusted p-value < 0.001. Significantly enriched pathways (adjusted p-value < 0.05) were selected and visually represented using *ggplot2.* Finally, to effectively illustrate the significance of the top hits derived from the list of differentially expressed genes, violin plots were employed as a visual tool.

## Results and discussion

### Tumor-induced shifts in LN stromal composition and transcriptome

Healthy tissue stroma is not thought to be inherently permissive to the growth of malignant cells, and the reprogramming of these tissues is believed to represent a requisite step to successful growth and progression of the tumor. Normal tissue fibroblasts, for instance, have long been known to exhibit cancer-restrictive characteristics, and it is therefore likely that the functional attributes of fibroblasts must first be modulated – coopted by the cancer to support rather than restrict growth and invasiveness. ^30^^–^^32^ The necessity of such microenvironmental reprogramming likely extends not only to the establishment of the primary tumor, but metastatic lesions as well. Successful engraftment of circulating cancer cells requires a supportive niche, and thus metastatic cancers must orchestrate a second iteration of this reprogramming process within distant tissues. ^8^^–^^13^

By design, lymph nodes are strategically positioned to intercept tissue-derived signals and antigen. Established tumors, however, appear to coopt the lymphatic infrastructure to facilitate metastatic dissemination. In this context, lymphatic drainage offers an efficient means of pre-conditioning tissue stroma for metastatic niche formation, and functions as a direct channel for the distribution of signals which may in fact subvert anti-tumor immunity. Indeed, efficiency of lymphatic drainage is positively correlated with metastatic potential, and lymphangiogenesis is a hallmark of metastatic niche formation. ^33^ In the pre-metastatic lymph node, expansion of both endothelium and FRCs has been documented.

**Figure 1.**
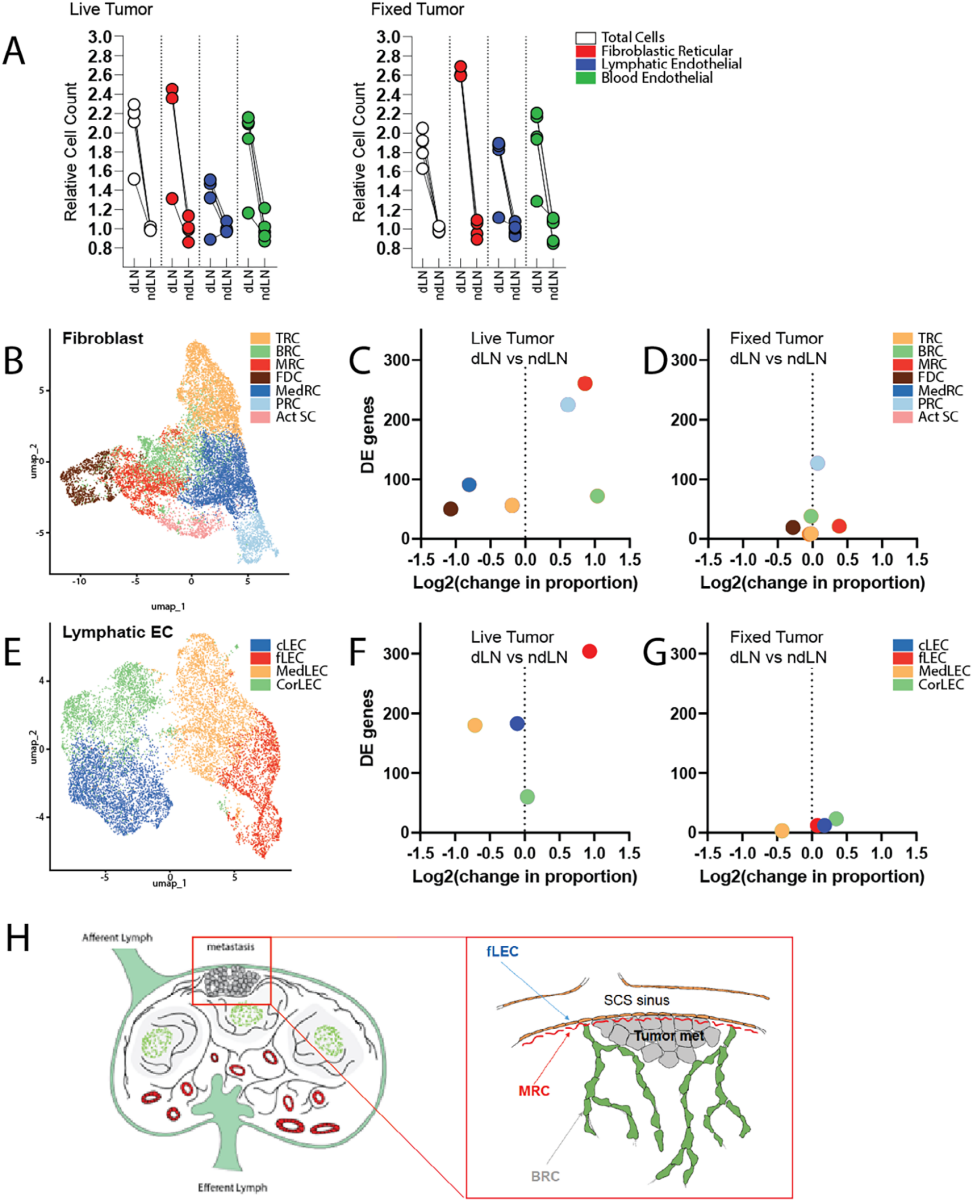
Single cell RNA sequencing of the tumor-draining lymph node: *Mice received either live or fixed MC38 cancer cells by subcutaneous injection.* *Tumor draining and contralateral non-draining inguinal lymph nodes were excised and enzymatically digested for analysis.* A) Quantification of cell numbers assessed by flow cytometry (n=5 mice per condition). B) UMAP visualization of total lymph node fibroblasts, colored by cluster identify. C, D) Quantification of the change in population proportion (amongst fibroblast subsets), along with total number of differentially expressed genes. *C.* dLN vs ndLN of mice growing live tumors. *D.* dLN vs ndLN of mice receiving fixed tumor cells. E) UMAP visualization of total lymph node lymphatic endothelial cells, colored by cluster identify. F, G) Quantification of the change in population proportion (amongst endothelial subsets), along with total number of differentially expressed genes. *F.* dLN vs ndLN of mice growing live tumors. *G.* dLN vs ndLN of mice receiving fixed tumor cells. H) Schematic representation of lymph node architecture and anticipated location of metastases engraftment. Inset depicts a micrometastasis surrounded by the following stromal subsets of interest: fLECs, MRCs, and BRCs.

However, stromal expansion in LNs is not a tumor-specific response program. Indeed, subcutaneous delivery of fixed tumor cells is sufficient to elicit LN swelling and expansion of FRCs, LECs, and BECs at a level comparable to that observed with live tumor growth ( Figure 1A). To explore more deeply the functional alterations occurring within the stromal compartment that specifically correlated with live tumor growth, we performed single cell RNA sequencing of cells isolated from draining and non-draining LNs in mice inoculated with either live or fixed MC38 cancer cells. We additionally enriched for PDPN-expressing LECs and FRCs to enable robust subset-level analyses. Within the resultant dataset, we identified 7 distinct clusters of fibroblastic cells and 4 clusters of lymphatic endothelial cells, each of which was annotated based on well-established identity markers ( Figure 1B, E). ^34^^–^^37^

The seeding of micrometastases within tumor-draining lymph nodes predictably occurs within the subcapsular sinus (SCS), where tissues are most readily exposed to lymph borne materials and migrating cancer cells ( Figure 1H). ^38^^,^^39^ Interestingly, we demonstrate here that unique cellular constituents of this specific microanatomical compartment were selectively impacted by the upstream tumor ( Figure 1C, F). Marginal reticular cells (MRCs) and floor lymphatic endothelial cells (fLECs) exhibited the greatest degree of transcriptional alteration in the tumor-draining LN relative to those of contralateral non-draining LNs. Perivascular cells (PRCs), though not localized to the SCS, likewise exhibited significant transcriptomic changes, which may reflect support of vascular expansion.

Unexpectedly, we also found that the overall cellular representation of these populations, along with B-zone reticular cells (BRCs), were disproportionately increased in the tumor-draining LN ( Figure 1C, F). This appears to differ from the pattern of stromal expansion that occurs in response to normal antigenic challenge. While exposure to fixed tumor cells elicited stromal activation and expansion – as previously demonstrated - this proliferative response seemed to occur proportionately amongst fibroblast and endothelial subsets and involved comparatively fewer transcriptomic alterations ( Figure 1D, G). Ultimately, we predict that selective induction of transcriptional changes and disproportionate expansion of stromal subsets comprising the SCS in the tdLN reflects the establishment of a niche supportive of metastatic engraftment and survival.

### MRC and BRCs exhibit elevated ECM production reminiscent of desmoplastic CAFs

**Figure 2.**
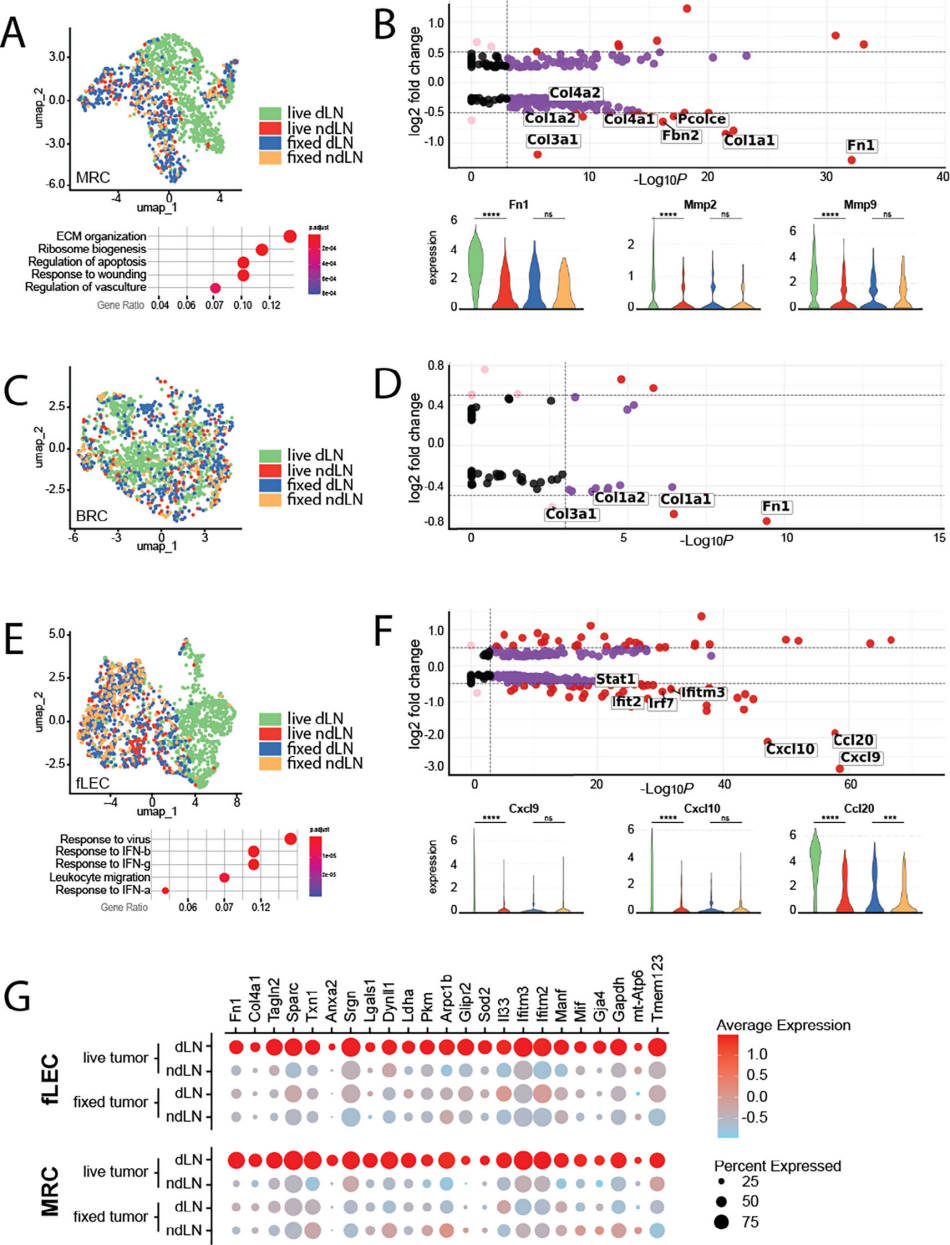
Transcriptomic features of MRCs, BRCs, and fLECs in the tumor-draining lymph node. A) ( *top*) UMAP visualization of MRCs, colored by treatment condition. ( *bottom*) Pathways significantly enriched in MRCs of live-tumor-draining LNs relative to non-draining LNs. B) ( *top*) Volcano plots of differentially expressed genes between MRCs of draining vs non-draining LNs. Select genes of interest are highlighted. ( *bottom*) Violin plots of select genes differentially expressed in MRCs of the live-tumor draining LN. Expression is shown across all four experimental conditions; live draining, live non-draining, fixed draining, and fixed non-draining. C) UMAP visualization of BRCs, colored by treatment condition. D) Volcano plots of differentially expressed genes between BRCs of draining vs non-draining LNs. Select genes of interest are highlighted. E) ( *top*) UMAP visualization of fLECs, colored by treatment condition. ( *bottom*) Pathways significantly enriched in fLECs of live-tumor-draining LNs relative to non-draining LNs. F) ( *top*) Volcano plots of differentially expressed genes between fLECs of draining vs non-draining LNs. Select genes of interest are highlighted. ( *bottom*) Violin plots of select genes differentially expressed in fLECs of the live-tumor draining LN. Expression is shown across all four experimental conditions; live draining, live non-draining, fixed draining, and fixed non-draining. G) Dotplot displaying expression of transcripts upregulated across both MRCs and fLECs in the live tdLN. The size of the dot represents percentage of cells expressing transcript, while the color indicates average level of expression within the population.

While the exact microenvironmental preferences of primary and secondary lesions may differ, and the means by which these alterations are driven may be distinct, there are almost certainly commonalities in tumor-induced stromal reprogramming. For instance, the aberrant production of extracellular matrix, characteristic of the desmoplastic response, is broadly observed across cancer indications. These responses are largely driven by conversion of resident fibroblast populations to tumor-supportive cancer associated fibroblasts (CAFs). Within the LN SCS, resident fibroblast populations include primarily MRCs that underly the sinus endothelium, and a proportion of BRCs that populate the interfollicular space. As both populations exhibited shifts specific to the tdLN, we re-clustered each population and performed differential transcriptomic analyses to identify tumor-specific changes ( Figure 2A-D). Amongst these changes, MRCs most notably exhibited increases in a broad spectrum of ECM components and matrix remodeling enzymes reminiscent of desmoplastic CAFs of the primary tumor, including upregulation of fibronectin, a critical ECM protein involved in development, wound healing, and cancer metastasis ( Figure 2A,B). ^40^ Interestingly, PDAC-derived exosomes were recently reported to induce elevated fibronectin expression in the pre-metastatic liver - a process which was found to be essential for niche formation. ^41^

In addition to ECM proteins, matrix metalloproteases (MMPs) likewise play a pivotal role in cancer progression and metastatic dissemination through matrix remodeling. ^42^^,^^43^ Along these lines, we observed increases in both MMP2 and MMP9 specifically in MRCs of the tdLN ( Figure 2B). While it is unclear from transcriptomic signature alone how the structure of the ECM is specifically remodeled in the SCS, or the extent to which this may impact the nascent metastasis, expression of both has previously been linked to niche formation and vascular remodeling in pre-metastatic tissues and are thus of particular interest. ^9^^,^^44^^,^^45^

Interestingly, previous reports have noted significant tumor-induced enlargement of the collagen-rich LN conduits – a branched network of fibers which normally functions as a transport system for small soluble materials through the LN cortex and paracortex. ^25^ In this context, Increased conduit thickness correlated with greater transport of larger, normally size-excluded molecules, and the authors speculated that these alterations may result in abnormal distribution of tumor-derived materials to deeper areas of the LN parenchyma. This is notably counter to the effects observed during acute inflammation-associated LN expansion. Under such conditions, LN conduits are also structurally disrupted, but size exclusivity of molecular transport through the network is nevertheless maintained. ^46^^,^^47^ This distinction is particularly intriguing, as size exclusivity of LN conduits is believed to be a function of plvap-lined gating channels that traverse the sinus-lining floor LECs (fLECs), rather than an intrinsic feature of conduit size itself. ^48^ Lymph-borne molecules small enough to pass through these trans-endothelial channels subsequently gain access to the conduit network. We thus speculate that matrisomal dysregulation surrounding fLECs and the underlying MRCs, such as those we find describe within this dataset, may in fact account for, or contribute to, this distinct loss of regulated conduit access in tdLNs.

BRCs likewise exhibited upregulation of some ECM-related transcripts in the tdLN, however the overall extent of differentially expressed genes in this subset was markedly less expansive ( Figure 2C, D). Nevertheless, we note that BRC representation within the dataset was significantly greater in the tdLN, possibly reflecting a greater proliferative response without significant functional divergence from baseline properties of BRCs in a resting LN. As BRCs are essential to the homeostatic maintenance of naïve B cells within the LN, proliferative expansion of the BRC subset may functionally mirror the known preferential accumulation of B cells in tumor draining nodes. ^49^^,^^50^ In turn, accumulation of B cells within the tdLN has been shown to drive pro-metastatic lymphangiogenesis, and thus expansion of the B cell support network may be an indirect, but requisite factor in support of this process. ^21^

### fLECs engage specific chemotactic cues within the premetastatic LN niche

Cell recruitment, migration and positioning into and within the LN is a highly regulated process, guided by an array of precisely coordinated chemotactic gradients. Principal among these chemotactic cues are the homeostatic chemokines Ccl19, Ccl21, and Cxcl13, each of which is primarily produced by subsets of LN FRCs. ^51^^–^^54^ Previous studies characterizing changes in the tdLN have highlighted a transcriptional downregulation of these cues within 3 days of tumor implantation, and subsequent loss of normal immune cell compartmentalization. Such perturbations would seemingly be detrimental to the initiation of adaptive immune responses, which depends on precise cellular positioning. Indeed, genetic mouse models of homeostatic chemokine disruption exhibit impaired immunity. However, it is notable that all forms of inflammation and antigenic challenge, not just exposure to tumor-derived factors, can trigger transient downregulation of these factors. ^55^^–^^61^ As such, the immunological consequences of chemokine downregulation following tumor inoculation remain unclear. Within our dataset, however, we did not observe significant transcriptional alterations to Ccl19, Ccl21, or Cxcl13 amongst any stromal subset (data not shown). This may be reflective of the later timepoint at which we collected our data, and the transient nature of these fluctuations.

While we do not observe dysregulation of these FRC-associated chemokines, re-clustering and differential analysis of the fLEC population did in fact reveal engagement of pathways related to leukocyte migration ( Figure 2E, F). In particular, we observed dramatic increases in the inflammation-associated chemokines Cxcl9, Cxcl10 and Ccl20 ( Figure 2F). These changes were exclusively depicted within the fLEC population, suggesting specific chemoattraction of cell types expressing the corresponding cognate receptors to the SCS. Interestingly, the Ccl20/Ccr6 axis has been well explored in the context of metastatic colorectal cancer, wherein CCr6-expressing cancer cells are recruited to the liver by Ccl20-expressing periportal stromal cells. ^62^ Expression of Ccl20 by floor LECs in the tdLN may be similarly utilized by migrating cancer cells in lymphatics. Critically, Ccl20 is absent on neighboring ceiling LECs, allowing the formation of a differential gradient of chemokine across the LN SCS. Analogous chemotactic gradients of Ccl19 and Ccl21 across the SCS have been experimentally demonstrated as critical for the recruitment of migrating dendritic cells into the LN. ^63^

By contrast, Cxcl9 and Cxcl10 are interferon induced chemokines that are most well described in the recruitment and activation of antigen experienced, Cxcr3-expressing T and NK cells. ^64^^,^^65^ Expression of these chemokines is therefore consistent with the broader interferon response signature of exhibited by fLECs of the tdLN ( Figure 2E). In the context of cancer, these chemokines are notable for their role in recruitment of tumor-infiltrating lymphocytes and are thought to be important for driving immunologically “hot” tumor phenotypes. ^66^ Expression of Cxcl9 and Cxcl10 within the LN is additionally known to support positioning of memory T cells to the interfollicular region and is critically important for antiviral immune responses. ^67^^,^^68^ Cumulatively, these observations may implicate expression of Cxcl9/Cxcl10 by fLECs as a beneficial means to direct antigen responsive immune cells to site of future metastatic engraftment. On the other hand, Cxcr3 may also be expressed by migrating cancer cells, and the Cxcl9/Cxcl10/Cxcr3 signaling axis has in fact been implicated in the growth and metastasis of tumors to various organs, including the LN. ^69^^–^^72^ Thus, it remains unclear whether increased expression of these chemokines in the premetastatic LN favor metastasis engraftment or anti-tumor immunity.

### Stromal expression ofIL33 is increased in the pre-metastatic niche

While MRCs and fLECs exhibited unique subset-specific responses in the tdLN, we also noted a handful of upregulated transcriptional elements common across both populations ( Figure 2G). Importantly, upregulation of each was observed only in the draining LN of mice bearing live tumors but did not occur in the LNs of mice inoculated with fixed tumor cells ( Figure 2G). As such, we believe these genes to be uniquely induced in response to tumor-derived signals.

Exposure to factors produced by an upstream tumor may elicit responses that skew immune education, and thus in this context, the striking increase in IL33 – a cytokine with numerous immunological effects that are both context and cell-type specific - was particularly intriguing. ^73^ Notably, IL33 is a known driver of ST2+ T-reg expansion and may have an important role in the induction of Treg-mediated tolerance. ^74^^–^^77^ In the context of cancer metastasis, ST2-expressing Tregs have been shown to support tumor development, and *in vivo* inhibition of IL-33 signaling has been shown to disrupt formation of metastases in mouse models. ^74^ Additionally, elevated IL33 expression has been described in fibroblasts of lung metastases, wherein signaling is thought to contribute to a supportive niche through skewing immunity towards a Th2 response. ^78^ Tumor-induced IL33 expression across stromal subsets in the LN SCS may therefore have important implications for suppression of anti-tumor responses. Along these lines, recent reports have indeed suggested that metastatic spread to the LN can elicit immunological tolerance through the preferential induction of antigen-specific Tregs. ^79^ The functional consequence of this redirected immune response is thus believed to be favorable for subsequent metastatic outgrowth in distant organs.

By contrast, it is also notable that, under infectious conditions, IL33 secretion by LN fibroblasts has been shown to be a stress response program critical for CD8 T cell immunity. ^80^ It is thus possible that IL33 upregulation in tdLNs may alternatively engage both anti-tumor immunity or immune suppressive elements, the balance of which may be a critical determinant of tumor progression and metastatic spread.

Interestingly, a recent report has also implicated IL-33 signaling as a driver of fibroblast-to-CAF differentiation in oral squamous cell carcinoma. ^81^ In this context, IL-33 was found to be upregulated and stabilized by signaling from a novel lncRNA (termed Lnc-CAF), which resulted in acquisition of an activated CAF phenotype and subsequently supported tumor growth. Critically, Lnc-CAF was found to be distributed via tumor cell-derived exosomes, offering a plausible means by which this pathway might mediate preconditioning of LN stroma. Cumulatively, these studies suggest that IL-33 may function not only as a modulator of immunity in the metastatic microenvironment, but as a driving factor in the stromal acquisition of CAF-like features and niche formation.

### Metabolic reprogramming in LN stroma

The upregulation of LDHA and PKM transcript was likewise common across both MRCs and fLECs ( Figure 2G). This is particularly noteworthy within the context of niche formation and metastasis, as both these genes encode metabolic enzymes associated with the switch to aerobic glycolysis and have long been associated with metabolic adaptation in cancer cells. ^82^^,^^83^ Preferential utilization of aerobic glycolysis, even in the presence of sufficient oxygen, is termed the Warburg Effect, and is characteristic of cancer cell metabolism. ^84^ Despite several decades of research, a full functional account of this metabolic preference remains incompletely understood. However, disruption of this process by targeting of LDHA drives beneficial anti-tumor effects. ^85^^–^^88^

Notably, Riedel *et al.* recently demonstrated that inhibition of LDHA could reverse tumor-directed FRC activation phenotypes, suggesting a putative metabolic switch in stroma that may be similar to that of cancer cells. ^24^ The authors proposed a model wherein elevated levels of lactate produced in the primary tumor drains to the sentinel lymph node, modulating FRC function and conditioning the pre-metastatic niche. Supportive of this hypothesis, they observed higher expression of LDHA near lymphatic vessels of the primary tumor, and elevated levels of lactate in the draining LN. By contrast, our data suggest that LDHA is in fact intrinsically upregulated in both MRCs and fLECs as well, potentially further contributing to the regional build-up of lactate.

Whether this response is secondary to the drainage of lactate from the primary tumor, or rather a response to other yet unidentified signals, remains unknown. However, a recent demonstration that CAFs from the primary tumor can in fact impart Warburg-like metabolism via exosomal transfer of metabolites offers an intriguing alternative whereby CAF-derived exosomes passing through lymphatic drainage may likewise contribute to induction of metabolic preconditioning at the site of future LN metastasis. ^89^ Nevertheless, both the means by which LDHA/PKM are upregulated and the metabolic and functional consequences of these alterations bear further exploration.

## Concluding remarks

Metastatic progression is the greatest cause of mortality across most cancer indications, and thus developing new means of clinical intervention in this process is clearly of paramount importance. We believe the findings discussed herein provide an important resource for understanding the early impact of cancer-associated signals on metastatic niche formation. Additionally, we provide comparative analysis against LN stroma following antigenic challenge with fixed cancer cells, allowing us to discern changes that relate to factors actively produced by tumors from generalized inflammatory response programs. Transcriptomic changes discussed herein thus represent tumor-specific responses that may prove to be attractive targets for preventative or therapeutic intervention. However, further experimental exploration will be required to establish causal relationships between these transcriptional changes and metastatic success rate. Additionally, the specific identity of secreted factors driving LN preconditioning are not explored in this study - though paired analyses of primary tumors and tdLN might prove informative in this regard. Ultimately, we suggest that continued exploration along these lines may critically enable the identification of novel pressure points against which transformative therapeutic or preventative intervention may be directed.

## Accession numbers

Gene Expression Omnibus at NCBI: Tumor-driven stromal reprogramming in the pre-metastatic lymph node [Mus musculus (house mouse)]. Accession number GSE248905; http://identifiers.org/geo: GSE248905

## Data Availability

Open Science Framework: Underlying data for ‘Tumor-driven stromal reprogramming in the pre-metastatic lymph node’, https://www.doi.org/10.17605/OSF.IO/WDV3H. ^27^ This project contains the following underlying data:
• Data File: Tumor dLN_A1_A01.fcs • Data File: Tumor dLN_A2_A02.fcs • Data File: Tumor dLN_A3_A03.fcs • Data File: Tumor dLN_A4_A04.fcs • Data File: Tumor dLN_A5_A05.fcs • Data File: Tumor NdLN_B1_B01.fcs • Data File: Tumor NdLN_B2_B02.fcs • Data File: Tumor NdLN_B3_B03.fcs • Data File: Tumor NdLN_B4_B04.fcs • Data File: Tumor NdLN_B5_B05.fcs • Data File: Killed dLN_C1_C01.fcs • Data File: Killed dLN_C2_C02.fcs • Data File: Killed dLN_C3_C03.fcs • Data File: Killed dLN_C4_C04.fcs • Data File: Killed dLN_C5_C05.fcs • Data File: Killed NdLN_D1_D01.fcs • Data File: Killed NdLN_D2_D02.fcs • Data File: Killed NdLN_D3_D03.fcs • Data File: Killed NdLN_D4_D04.fcs • Data File: Killed NdLN_D5_D05.fcs • Mouse FCS data key.xlsArrive Author Checklist.pdf Open Science Framework: ARRIVE checklist for ‘Tumor-driven stromal reprogramming in the pre-metastatic lymph node’, https://www.doi.org/10.17605/OSF.IO/WDV3H. ^27^ Data are available under the terms of the Creative Commons Zero “No rights reserved” data waiver (CC0 1.0 Public domain dedication).
