# Supplementary material for: Tumor-driven stromal reprogramming in the pre-metastatic lymph node
Source: F1000Res. 2025 Oct 7;13:223. Originally published 2024 Mar 27. [Version 3] doi: 10.12688/f1000research.145171.3 (PMC12547382; doi:10.12688/f1000research.145171.3)
Supplement: Peer review history

# Reviewer report: Reviewer response for version 3

**DOI:** 10.5256/f1000research.188508.r421293

**Published:** 2025-10-22

**Authors:** Sanjiv Luther, Marija Jokic

**Affiliations:** 1 Department of Immunobiology, University of Lausanne, Lausanne, Switzerland; 2 Immunobiology, University of Lausanne, Epalinges, Vaud, Switzerland

I am satisfied with the changes made. I have no further comments.

Is the work clearly and accurately presented and does it cite the current literature?

Partly

If applicable, is the statistical analysis and its interpretation appropriate?

Yes

Are all the source data underlying the results available to ensure full reproducibility?

Yes

Is the study design appropriate and is the work technically sound?

Yes

Are the conclusions drawn adequately supported by the results?

Partly

Are sufficient details of methods and analysis provided to allow replication by others?

Yes

Reviewer Expertise:

lymph nodes, stromal cells, immunity, scRNAseq

We confirm that we have read this submission and believe that we have an appropriate level of expertise to confirm that it is of an acceptable scientific standard.

# Reviewer report: Reviewer response for version 2

**DOI:** 10.5256/f1000research.178762.r377193

**Published:** 2025-05-10

**Authors:** Sanjiv Luther

**Affiliations:** 1 Department of Immunobiology, University of Lausanne, Lausanne, Switzerland

The authors have made major changes in response to my comments by adding supplementary figures and tables that help a lot in reading and understanding the manuscript. The new caveat section is also helpful given that this study is limited to the scRNAseq datasets described.

 Minor points:

1. Typo in figure 1 E legend: colored by cluster identity ? or a function ‘cluster identify’ and put in italic?
2. Supplementary figure 2 legend: say ‘pooled’ fibroblast or ‘pooled’ endothelial UMAP to be clearer?
3. Supplementary table 3 legend: correct ‘figure 2B,D,F’
4. Page 9: …chemotactic gradients of CCL19: the authors of cited ref 67 showed nothing with CCL19 but probably another paper should have been cited instead showing an ACKR4 role in DC trafficking from the SCS into the T zone due to CCL21 gradient shaping (Ulvmar, Immunity 2014). That paper did not show CCL19 gradient shaping by ACKR4 in vivo but provided only in vitro evidence; please adapt the claim accordingly.
5. Reply to major comment 1: Madcam1 transcript expression is being used as marker to identify MRC / FDC versus Madcam1-negative BRC, with reference to previous studies looking at LNs in homeostasis or viral infection (refs 33 and 34). While ref 33 shows that Madcam1 transcript levels are a poorly reliable subset marker, ref 34 indicates Madcam1 expression being also present in non-MRC/FDC, although at lower levels than in the latter. In the current study, the authors look at LN stroma in a different setting (tumor drainage and antitumor immunity) not previously described by scRNAseq, with Madcam1 being potentially upregulated upon stromal cell activation (by signals like LTab, similar to LN development). Thus, BRC other than MRC/FDC may express or even upregulate Madcam1 in this setting and thus be misclassified in the current study. The use of few and variable subset markers like Madcam1 is a caveat of the present study given that the authors do not provide data to confirm their claims at the protein and histological level. I recommend that the authors include this point in the caveat section of this manuscript.
6. Page 11 (and reply to major comment 4): Tumor cells were indirectly identified among Pdpn-CD31- cells as being PDL1+. This seems a very vague identification that may be considerably contaminated by various Pdpn-CD31- fibroblast subsets that are likely to upregulate PDL1 upon IFN sensing. Thus, this supplementary figure 3 along with the text should only be shown if the authors have solid evidence for these cells being strongly enriched in metastatic tumor cells.
7. Please verify validity of accession number for the identifiers.org database.

Is the work clearly and accurately presented and does it cite the current literature?

Partly

If applicable, is the statistical analysis and its interpretation appropriate?

Yes

Are all the source data underlying the results available to ensure full reproducibility?

Yes

Is the study design appropriate and is the work technically sound?

Yes

Are the conclusions drawn adequately supported by the results?

Partly

Are sufficient details of methods and analysis provided to allow replication by others?

Yes

Reviewer Expertise:

lymph nodes, stromal cells, immunity, scRNAseq

I confirm that I have read this submission and believe that I have an appropriate level of expertise to confirm that it is of an acceptable scientific standard, however I have significant reservations, as outlined above.

# Response

**Published:** 2025-09-23

**Authors:** Jonathan Chang

**Affiliations:** Oncology, Novartis, Cambridge, MA, USA

**Response to Reviewer's comments Below:**

*Reviewer Comment 1:  Typo in figure 1 E legend: colored by cluster identity ? or a function ‘cluster identify’ and put in italic?* **Response:  Typo corrected**

*Reviewer Comment 2: Supplementary figure 2 legend: say ‘pooled’ fibroblast or ‘pooled’ endothelial UMAP to be clearer?* **Response: Changed according to reviewer’s suggestion.**

*Reviewer Comment 3: Supplementary table 3 legend: correct ‘figure* *2B,D,F’.* **Response** **: legend has been corrected**

*Reviewer Comment 4: Page 9: …chemotactic gradients of CCL19: the authors of cited ref 67 showed nothing with CCL19 but probably another paper should have been cited instead showing an ACKR4 role in DC trafficking from the SCS into the T zone due to CCL21 gradient shaping (Ulvmar, Immunity 2014). That paper did not show CCL19 gradient shaping by ACKR4 in vivo but provided only in vitro evidence; please adapt the claim accordingly.* **Response** *:* **Thank you for your suggestion – we believe the currently cited paper from Ulvmar *et al *(Nat Imm 2014) is still appropriate for the intended claim.  In the cited paper, the authors demonstrate 1) Ccl21 gradient across the SCS is shaped by cLEC expression of ACKR4 (aka *CCLR1*), 2) loss of ACKR4 impacts DC emigration to the LN from the lymphatics *in vivo*, and 3) ACKR4 could create functional gradients of Ccl19 *in vitro.  *You are correct that they did not specifically demonstrate Ccl19 gradients across the SCS *in vivo*, and this is largely because Ccl19 is soluble and cannot be readily imaged in tissues.  However, because ACKR4 scavenges both Ccl19/Ccl21, and both chemokines are similarly expressed in the SCS, the authors speculate that ACKR4 influences the patterning of both chemokines.  They state:  “Thus, we can only speculate that CCL19, produced at the same sites as CCL21, as well as potentially in the SCS itself, might be 'patterned' by CCRL1 on cLECs to form gradients similar to those of CCL21.”  Given the lack of explicit *in vivo *evidence regarding Ccl19 patterning, however, we will have slightly amended our original statement to reference only Ccl21 patterning.  The statement in question now reads “Analogous chemotactic gradients of Ccl21 across the SCS has been experimentally demonstrated as critical for the recruitment of migrating dendritic cells into the LN.”**

*Reviewer Comment 5:  (Reply to major comment 1): Madcam1 transcript expression is being used as marker to identify MRC / FDC versus Madcam1-negative BRC, with reference to previous studies looking at LNs in homeostasis or viral infection (refs 33 and 34). While ref 33 shows that Madcam1 transcript levels are a poorly reliable subset marker, ref 34 indicates Madcam1 expression being also present in non-MRC/FDC, although at lower levels than in the latter. In the current study, the authors look at LN stroma in a different setting (tumor drainage and antitumor immunity) not previously described by scRNAseq, with Madcam1 being potentially upregulated upon stromal cell activation (by signals like LTab, similar to LN development). Thus, BRC other than MRC/FDC may express or even upregulate Madcam1 in this setting and thus be misclassified in the current study. The use of few and variable subset markers like Madcam1 is a caveat of the present study given that the authors do not provide data to confirm their claims at the protein and histological level. I recommend that the authors include this point in the caveat section of this manuscript.* **Response:  We appreciate the careful consideration of *Madcam1* expression, and we agree that we cannot rule out the possibility of tumor-derived signals eliciting upregulation or downregulation of cluster-identification markers (whether *Madcam1* or any other marker).  As you note, there is some discrepancy between references 33 and 34 (Pikor *et al* and Rodda *et al*) with regard to *Madcam1* expression.  Notably, Pikor *et al *(Nat Imm 2020) acknowledged the discrepancy in *Madcam1* expression with that seen by Rodda *et al *(2018), and mentioned that their dataset resolved two tnfsf11+ subsets – one of which is *Madcam1*-positive, and the other which is *Madcam1*-negative.  It is therefore also possible that the difference between these two referenced papers relates to the resolution of clustering.  However, we would also like to emphasize that beyond *Madcam1* expression, specific expression of *tnfsf11* and elevated *enpp2* were also used as markers to help distinguish the MRC cluster.  Nevertheless, we do acknowledge that this study includes only transcriptomic data and relies on previously published studies to interpret subset identification.  And as noted by the reviewer, these previously published studies do not include an investigation of cluster identification within the context of a tumor-draining lymph node – as such we have amended the manuscript to include mention of this concern within the “Caveats and study limitations” section.**

*Reviewer Comment 6:  Page 11 (and reply to major comment 4): Tumor cells were indirectly identified among Pdpn-CD31- cells as being PDL1+. This seems a very vague identification that may be considerably contaminated by various Pdpn-CD31- fibroblast subsets that are likely to upregulate PDL1 upon IFN sensing. Thus, this supplementary figure 3 along with the text should only be shown if the authors have solid evidence for these cells being strongly enriched in metastatic tumor cells.* **Response** ***:  *The reviewer is correct that we cannot rule out the possibility of this population (PDPN-CD31-PDL1+) containing non-tumor cells.  In the amended manuscript, we have included mention of the possibility of PDPN-CD31- stromal cells upregulating PD-L1.  This supplementary figure was included in response to the reviewer’s previous question about the possibility of active metastatic growth in the tdLN.  As noted in our previous response, this population amounted to an average of just 114 total cells in the tdLN.  Whether this PDPN-CD31-PDL1+ population purely consists of mestatic tumor cells, or additionally contains activated DN stromal cells that have upregulated PD-L1, we feel that the conclusion should still be that there are very few (if any) tumor cells within the LN at time of collection.**

*Reviewer Comment 7:  Please verify validity of accession number for the identifiers.org database*. **Response:  The accession number is correct.  However there appears to have been a space incorrectly placed within the URL.  The correct URL is http://identifiers.org/geo:GSE248905 and this has been updated in the revised manuscript.**

# Reviewer report: Reviewer response for version 2

**DOI:** 10.5256/f1000research.178762.r377192

**Published:** 2025-04-26

**Authors:** Christopher McGinnis

**Affiliations:** 1 Department of Pathology, Stanford University, Gladstone-UCSF Institute of Genomic Immunology, Parker Institute for Cancer Immunotherapy, San Francisco, USA

The authors satisfactorily addressed all of my major and minor comments (with the exclusion of the handling of cell-cell doublets, but this does not detract from the overall claims of the manuscript).

Is the work clearly and accurately presented and does it cite the current literature?

Partly

If applicable, is the statistical analysis and its interpretation appropriate?

Partly

Are all the source data underlying the results available to ensure full reproducibility?

Yes

Is the study design appropriate and is the work technically sound?

Partly

Are the conclusions drawn adequately supported by the results?

Partly

Are sufficient details of methods and analysis provided to allow replication by others?

Partly

Reviewer Expertise:

Cancer immunology, metastasis biology, single-cell genomics

I confirm that I have read this submission and believe that I have an appropriate level of expertise to confirm that it is of an acceptable scientific standard.

# Reviewer report: Reviewer response for version 1

**DOI:** 10.5256/f1000research.159080.r296538

**Published:** 2024-09-14

**Authors:** Christopher McGinnis

**Affiliations:** 1 Department of Pathology, Stanford University, Gladstone-UCSF Institute of Genomic Immunology, Parker Institute for Cancer Immunotherapy, San Francisco, USA

In this study by Piquet et al, the Authors use single-cell RNA-sequencing (scRNA-seq) to compare lymph node (LN) stromal cell transcriptional profiles in mice bearing live and fixed tumors to disentangle changes in LN stromal cells related to pro-metastatic remodeling mechanisms from changes linked to antigenic challenge. The authors make the following claims:

  

1. Development of the LN pre-metastatic niche involves changes in the proportions and gene expression profiles of specific subtypes of fibroblasts and endothelial cells comprising the subcapsular sinus, in addition to other LN cell populations (e.g., b-zone reticular cells and perivascular cells).
2. MRCs in the live tdLN are induced into a desmoplastic CAF-like state associated with upregulation of ECM components and remodeling genes such as fibronectin and Mmp2/9. Authors speculate that tumor-reprogrammed MRCs contribute to altered regulation of LN conduit access.
3. fLECs increase expression of pro-inflammatory cytokines (e.g., Ccl20 and Cxcl9/10) in tdLNs, raising the question of whether TILs/NK cells or Cxcr3+ metastatic tumor cells (or both) are preferentially recruited to the subcapsular sinus.
4. MRCs and fLECs enact a coordinate gene expression program in the live tdLN associated with increased Il33 and aerobic glycolysis genes, providing new insights into stromal cell signaling and metabolism mechanisms in the LN metastatic niche.

Major Comments

  

1. Do the authors have evidence showing that mice harboring MC38 tumors actually form LN metastases? Or at least what proportion of the mice form mets? The MC38 tumor model is not thought to robustly metastasize (e.g., only ~50% of orthotopically-transplanted tumors form LN metastases; Greenlee & King, 2022), especially after subcutaneous injection. Thus, it is unclear whether the signatures described in the paper truly represent the pre-metastatic niche LN. Without adequately addressing this concern, the authors need to recontextualize their observations to focus on the effects of primary tumor-mediated reprogramming on the tdLN rather than the pre-metastatic niche.
2.  “Within the resultant dataset, we identified 7 distinct clusters of fibroblastic cells and 4 clusters of lymphatic endothelial cells, each of which was annotated based on well-established identity markers” – Authors should include either reference to which markers were used or, better yet, a heatmap or dotplot showing expression levels for marker genes in each annotated cell type.
3. “Perivascular cells (PRCs), though not localized to the SCS, likewise exhibited significant transcriptomic changes, which may reflect support of vascular expansion” – While entirely possible, it is unclear how the observed increase in the numbers of DEGs suggests support of vascular expansion. Authors should analyze more deeply the genes that are differentially expressed (e.g., GSEA, targeted analysis of known proangiogenic factors expressed by PRCs, etc.) before making this claim.  
4. The Authors make many claims throughout the manuscript about proliferative expansion of certain fibroblast and endothelial cell subsets in tumor-draining lymph nodes, but do not show any direct evidence of this increased proliferation. Since annotating proliferative cells via Mki67 and Hells expression in scRNA-seq data is possible, I recommend the Authors compare the proportions of proliferative subsets in all experimental groups to test/strengthen these claims.
5. The Authors do not describe how they handled the removal of doublets during scRNA-seq analysis. The presence of doublets could significantly confound data interpretation – particularly in instances where the underlying cell type distributions between samples are known to differ, as in this case – and, thus, needs to be addressed to ensure the observations are not artefactual in nature.
6. In the Methods section, the Authors state that “The present study used tissues collected from naïve mice (n=4), mice bearing live MC38 tumors (n=3), or fixed MC38 tumor cells (n=3)” and then “No animals were excluded from analysis.” However, the described scRNA-seq analyses do not incorporate insights gained from the naïve mouse samples. Comparing naïve and fixed tumor samples could provide key insight for distinguishing the effects of live tumors on the tdLN. Moreover, including comparisons between naïve and fixed tumor samples would be critical for pinpointing the observed effects that are specifically due to antigenic challenge. Alternatively, if naïve samples were not used, the Methods section should be edited to clarify this point.
7. In Fig. 2G, the authors show genes that are specifically enriched in tdLN MRCs and fLECs. How were these genes identified? Is Il33 expression between tdLNs isolated from mice harboring live and fixed tumors statistically-significantly different? The up-regulation between tdLN and ndLN is clear in both live and fixed tumor settings, but tdLNs in fixed tumor samples also increase expression of Il33 compared to ndLNs, suggesting that this observation may not be live tumor-specific.  

Minor Comments

  

1. Authors state “In the pre-metastatic lymph node, expansion of both endothelium and FRCs has been documented” – true, but authors should cite appropriate literature references.
2. How did authors verify that MC38 tumor cells were successfully fixed after 4% PFA exposure?
3. Fig. 1A: Authors should clarify in the legend the reference point they used to calculate relative cell count. Imagining it was the average of the non-draining LN, but should be explicit to avoid confusion. Authors should also clarify how each cell type was identified using their flow cytometry panel.
4. Fig. 1B and E: Authors do not ever define some of the acronyms used here (e.g., TRC, FDC)
5. Page 7: “In this context, Increased conduit thickness…” – ‘Increased’ should not be capitalized.
6. Fig. 2B/E/F – authors should be explicit about what the volcano plot colors scheme to ensure clarity of interpretation. Also, are the p-values presented raw or adjusted?
7. As far as I can tell, the Authors use increased expression of fibronectin by MRCs in the tdLN to support the claim that they are induced into a desmoplastic CAF-like state. This may indeed be the case, but more thorough analyses/discussions are needed to sufficiently support this claim. I would suggest leveraging publicly-available scRNA-seq data of desmoplastic CAFs (if such data exists) to assess similarly in transcriptional signatures. Alternatively, the language can be edited to lessen the claimed connection.
8. “Indeed, genetic mouse models of homeostatic chemokine disruption exhibit impaired immunity.” Need reference.
9. Should correctly note gene/protein identifiers (e.g., italics for gene names, proteins capitalized, etc.).
10. The 30% mitochondrial gene expression threshold is quite high. Authors should interrogate whether any high pMito clusters were retained in analyses that could confound interpretation.

On balance, I believe that the presented work represents a useful advance for the field, and I recommend its indexing, assuming that the concerns addressed above are satisfactorily addressed. Moreover, I would also like to specifically note that the manuscript is extremely well-written.

Is the work clearly and accurately presented and does it cite the current literature?

Partly

If applicable, is the statistical analysis and its interpretation appropriate?

Partly

Are all the source data underlying the results available to ensure full reproducibility?

Yes

Is the study design appropriate and is the work technically sound?

Partly

Are the conclusions drawn adequately supported by the results?

Partly

Are sufficient details of methods and analysis provided to allow replication by others?

Partly

Reviewer Expertise:

Cancer immunology, metastasis biology, single-cell genomics

I confirm that I have read this submission and believe that I have an appropriate level of expertise to confirm that it is of an acceptable scientific standard, however I have significant reservations, as outlined above.

# Response

**Published:** 2025-03-28

**Authors:** Jonathan Chang

**Affiliations:** Oncology, Novartis, Cambridge, MA, USA

**(Major) Reviewer Comment 1:**  Do the authors have evidence showing that mice harboring MC38 tumors actually form LN metastases? Or at least what proportion of the mice form mets? The MC38 tumor model is not thought to robustly metastasize (e.g., only ~50% of orthotopically-transplanted tumors form LN metastases; Greenlee & King, 2022), especially after subcutaneous injection. Thus, it is unclear whether the signatures described in the paper truly represent the pre-metastatic niche LN. Without adequately addressing this concern, the authors need to recontextualize their observations to focus on the effects of primary tumor-mediated reprogramming on the tdLN rather than the pre-metastatic niche. **Response: ** The reviewer raises a salient point in that subcutaneously implanted tumor models rarely in fact metastasize (at least within the timeframe at which the primary tumor remains within ethical growth limits).  Orthotopically implanted tumors more readily metastasize, perhaps due to the native regional tissue environment of implantation.  However, such models are often technically challenging and highly variable in terms of tumor growth kinetics.  We opted to use subcutaneous MC38 tumors to minimize technical and experimental variance which might otherwise obscure the identification of transcriptional signatures influenced by tumor-derived factors.  We feel the nature of multiple stromal alterations highlighted in this paper align well with previously proposed microenvironmental dependencies for tumor growth and metastasis highlighted within the broader literature, and it is for this reason that we contextualize these changes as “pre-metastatic”.  However, we fully recognize that data herein do not constitute experimental proof that any or all of these transcriptional changes are in fact necessary for metastasis but rather offer these results as a resource for more definitive studies to follow.  We agree that such experimental proof would necessitate the use of models for which lymph node metastasis can be explicitly documented following experimental perturbation.  Such an effort is currently beyond the scope of this limited study.  However, we do acknowledge that this is a critically important limitation, and discussion of this point has been included in the revised manuscript under the subheading “Caveats and study limitations”.

**(Major) Reviewer Comment 2:** “Within the resultant dataset, we identified 7 distinct clusters of fibroblastic cells and 4 clusters of lymphatic endothelial cells, each of which was annotated based on well-established identity markers” – Authors should include either reference to which markers were used or, better yet, a heatmap or dotplot showing expression levels for marker genes in each annotated cell type. **Response: **Cluster identification for our dataset was based on findings from previously published studies that have deeply characterized of stromal subsets (utilizing combined scRNAseq and IHC approaches of dissociated and intact lymph nodes respectively).  We aligned expression patterns of the markers validated in these studies with differential expression patterns of our identified clusters to assign subtypes.  Notable markers used for cluster identification are noted below, and bubble plots for each fibroblastic or endothelial cluster are now included in Supplementary Fig 1 of the revised manuscript as well as the methods section (these Key identifiers also listed below).  Additionally, DEG lists were included in Supplementary table 1 (for fibroblast subclusters) and Supplementary table 2 (for endothelial subclusters). Lymphatic Endothelial Subsets of the LN were annotated based on characterization work from den Braanker, *et al. *2021 (PMID: 34769408) and Fujimoto, *et al.* 2020 (PMID: 32251437).  Key identifiers for each subset include the following: **cLEC**: *ackr4+, cd36+, edn1+, anxa2+*. **fLEC**: *lyve1+, glycam1+, coch+, madcam1+* **Medullary LEC**: *lyve1+, marco+, il33+, itg2b+* **Cortical LEC**: *mrc1+, ptx3+, reln+*. Fibroblastic Subsets of the LN were annotated based on characterization collective work from multiple studies including Rodda, *et al. *2018 (PMID: 29752062), Pikor *et al.* 2021 (PMID: 32424359) and Beuchler *et al.* 2021 (PMID: 33981032).  Key identifiers for each subset include the following: **TRC**: *ccl19+, ccl21+, grem1+* **BRC**: *cxcl13+, cr2-, madcam1-, tnfsf11-, ch25h+* **FDC**: *cxcl13+, madcam1+, coch+, cr2+, tmem119+, pthlh+* **MRC**: *madcam1+, tnfsf11+, enpp2+* **MedRC**: *cxcl12(hi), inmt+, stc1+, bst1-* **PRC**: *cd34+, fndc1+, pi16+, dpp4+* **Activated SC**: *nr4a1+.*

**(Major) Reviewer Comment 3:**  “Perivascular cells (PRCs), though not localized to the SCS, likewise exhibited significant transcriptomic changes, which may reflect support of vascular expansion” – While entirely possible, it is unclear how the observed increase in the numbers of DEGs suggests support of vascular expansion. Authors should analyze more deeply the genes that are differentially expressed (e.g., GSEA, targeted analysis of known proangiogenic factors expressed by PRCs, etc.) before making this claim. **Response: **This was a speculative comment due to the association of PRCs with LN vasculature, and the observed increase in BEC proliferation (fig1A). We have tempered this statement in the revised manuscript and included references to studies outlining potential function of PRCs.  Importantly, we have also revised our discussion of PRC, in which we describe them as “not localized to the SCS”.  Further review of the findings by Rodda *et al. *2018 in which they describe localization of CD34+ stromal cells not only in the perivascular compartment, but also within the LN capsule.  Transcriptional features identifying our PRC subset align with these CD34 SCs described by Rodda *et al.* and Sitnik *et al. *2016.   We have additionally included the full DEG list of PRCs in tdLN vs ndLN in the newly added Supplementary table 3. 

**(Major) Reviewer Comment 4:**  The Authors make many claims throughout the manuscript about proliferative expansion of certain fibroblast and endothelial cell subsets in tumor-draining lymph nodes, but do not show any direct evidence of this increased proliferation. Since annotating proliferative cells via Mki67 and Hells expression in scRNA-seq data is possible, I recommend the Authors compare the proportions of proliferative subsets in all experimental groups to test/strengthen these claims. **Response: **Increased cell counts of each major stromal cell population (fibroblast, LEC, and BEC) within the dLN vs ndLN (seen in Fig1A) provides us some direct evidence of proliferative expansion of each of these broader populations.  Within each stromal cell type, we then identify changes in the representation of subsets within in the scRNAseq dataset.  For instance, “Fibroblasts” are presumed to have proliferated due to the >2 fold increase in cell numbers relative to matched non-draining LN (fig1A).  Then, within all fibroblasts, there is then a higher proportion of MRC, PRC, and BRC in the dLN relative to the ndLN (Fig 1C), a differential which is not present in the control conditions of mice inoculated with fixed tumor cells (Fig 1D).  This differential in subset representation is also visually evident in the newly added Supplementary figure 2.  Unfortunately we do not have the capacity to measure absolute cell counts of individual subsets, and thus proliferative expansion is inferred from the combination of these above observations.  Additionally, *ki67*+ cells are indeed present in our dataset, but constitute a small proportion of every cell population present.  In our experience, *ki67* has rarely been a reliable or quantitative measure of population expansion in tissues.

**(Major) Reviewer Comment 5:**  The Authors do not describe how they handled the removal of doublets during scRNA-seq analysis. The presence of doublets could significantly confound data interpretation – particularly in instances where the underlying cell type distributions between samples are known to differ, as in this case – and, thus, needs to be addressed to ensure the observations are not artefactual in nature. **Response: **We carefully assessed the quality control metrics of the dataset, including the number of RNA molecules (UMIs) and the number of genes detected per cell. All values fell within the expected range for single cells, as defined in Seurat’s documentation, and we did not observe any anomalies indicative of doublets. Based on these observations, we do not suspect the presence of doublets in our dataset at a level that would confound the interpretation of our results. This information has been added to the methods description in the revised manuscript.

**(Major) Reviewer Comment 6:**  In the Methods section, the Authors state that “The present study used tissues collected from naïve mice (n=4), mice bearing live MC38 tumors (n=3), or fixed MC38 tumor cells (n=3)” and then “No animals were excluded from analysis.” However, the described scRNA-seq analyses do not incorporate insights gained from the naïve mouse samples. Comparing naïve and fixed tumor samples could provide key insight for distinguishing the effects of live tumors on the tdLN. Moreover, including comparisons between naïve and fixed tumor samples would be critical for pinpointing the observed effects that are specifically due to antigenic challenge. Alternatively, if naïve samples were not used, the Methods section should be edited to clarify this point. **Response: **We appreciate the careful review and thoughtful suggestion.  However naïve samples were not included in this assessment.  The methods section was corrected to reflect the proper cohort used in this analysis.

**(Major) Reviewer Comment 7:**  In Fig. 2G, the authors show genes that are specifically enriched in tdLN MRCs and fLECs. How were these genes identified? Is Il33 expression between tdLNs isolated from mice harboring live and fixed tumors statistically-significantly different? The up-regulation between tdLN and ndLN is clear in both live and fixed tumor settings, but tdLNs in fixed tumor samples also increase expression of Il33 compared to ndLNs, suggesting that this observation may not be live tumor-specific. **Response: **The genes in Fig. 2G were identified by performing DEG analysis across dLN and ndLN of the live and fixed tumor condition.  Genes which were commonly upregulated in both the MRC and fLEC of specifically the live tdLN subset were graphed in the bubble plot.  The mean expression of IL33 in the dLN of mice receiving fixed tumor cells is overall slightly elevated over the corresponding ndLN, but did not reach statistical significance.  A statistically significant change in expression was only seen in the dLN vs ndLN of live tumor-bearing mice. 

**(Minor) Reviewer Comment 1:**  Authors state “In the pre-metastatic lymph node, expansion of both endothelium and FRCs has been documented” – true, but authors should cite appropriate literature references. **Response: **Citation has been added.

**(Minor) Reviewer Comment 2:**  How did authors verify that MC38 tumor cells were successfully fixed after 4% PFA exposure? **Response: **No tumor growth was observed in mice receiving fixed tumor cells at the experimental endpoint.

**(Minor) Reviewer Comment 3:**  Fig. 1A: Authors should clarify in the legend the reference point they used to calculate relative cell count. Imagining it was the average of the non-draining LN, but should be explicit to avoid confusion. Authors should also clarify how each cell type was identified using their flow cytometry panel. **Response: **Cell counts are presented as relative to the average of corresponding non-draining lymph nodes.  Cell populations were identified by flow cytometry with the following markers:  Fibroblastic Reticular (CD45-CD31-PDPN+), Lymphatic Endothelial (CD45-, CD31+, PDPN+), and Blood Endothelial (CD45-, CD31+, PDPN-).  This information has been added to the figure legend in the revised manuscript.

**(Minor) Reviewer Comment 4:**  Fig. 1B and E: Authors do not ever define some of the acronyms used here (e.g., TRC, FDC) **Response: **The following definitions have been added to the text:  Fibroblastic populations include T-zone Reticular Cells (TRC), B zone Reticular cells (BRC), Marginal Zone Reticular Cells (MRC), Follicular Dendritic Cells (FDC), Medulary Reticular cells (MedRC), Perivascular Reticular cells (PRC), and activated stromal cells (Act SC).  Endothelial cell subsets include ceiling LECs (cLEC), floor LECs (fLEC), Medulary LECs (MedLEC), and Cortical LECs (CorLEC). 

**(Minor) Reviewer Comment 5:**  Page 7: “In this context, Increased conduit thickness…” – ‘Increased’ should not be capitalized. **Response: **Typo has been corrected

**(Minor) Reviewer Comment 6:**  Fig. 2B/E/F – authors should be explicit about what the volcano plot colors scheme to ensure clarity of interpretation. Also, are the p-values presented raw or adjusted? **Response: **The cutoffs for highlighted genes include a log2 fold change of 0.5 or above (horizontal dashed lines) and adjusted p-value above 0.001 (indicated by the vertical dashed lines).  This description is included in the figure legend of the revised manuscript.

**(Minor) Reviewer Comment 7:**  As far as I can tell, the Authors use increased expression of fibronectin by MRCs in the tdLN to support the claim that they are induced into a desmoplastic CAF-like state. This may indeed be the case, but more thorough analyses/discussions are needed to sufficiently support this claim. I would suggest leveraging publicly-available scRNA-seq data of desmoplastic CAFs (if such data exists) to assess similarly in transcriptional signatures. Alternatively, the language can be edited to lessen the claimed connection. **Response: **While we do single-out Fn1 in the text, we note that this is one of several genes differentially expressed in MRCs of the tumor dLN that relate to matrix deposition or tissue remodeling.  Many others are additionally labeled in figure 2b. Additionally, both ECM organization and wound-healing are prominent in the pathway analysis as shown in Fig2a.  For additional clarity, we have also included the full DEG list of MRC, BRC, fLEC, and PRC populations in the tdLN vs ndLN in Supplementary table 3 of the revised manuscript. 

 We feel comfortable drawing the comparison to desmoplastic CAFs given that these indicators of altered matrix/remodeling arise within the context of the tumor-draining LN.  However, to soften the claim, we have changed the wording of the section title from “reminiscent of desmoplastic CAFs” to instead read “reminiscent of the desmoplastic nature of CAFs”, as we acknowledge that ECM production/tissue remodeling are only one facet of CAF function.  We do not want to make the claim that the transcriptional changes we see indicate that MRCs have become CAFs, but rather that they have taken on a key function typically associated with CAFs.

**(Minor) Reviewer Comment 8:**  “Indeed, genetic mouse models of homeostatic chemokine disruption exhibit impaired immunity.” Need reference. **Response: **Relevant references have been added.

**(Minor) Reviewer Comment 9:**  Should correctly note gene/protein identifiers (e.g., italics for gene names, proteins capitalized, etc.). **Response:** We have reformatted gene/protein names to match convention.

**(Minor) Reviewer Comment 10:**  The 30% mitochondrial gene expression threshold is quite high. Authors should interrogate whether any high pMito clusters were retained in analyses that could confound interpretation. **Response: **We carefully examined the distribution of mitochondrial gene expression across cells and found no evidence of high mitochondrial clusters that would indicate stressed or damaged cells. Additionally, downstream analyses, such as clustering and differential expression, did not reveal any confounding effects that could be attributed to mitochondrial gene expression. Based on these observations, we are confident that the retained cells are biologically relevant and do not introduce bias into our results.

# Reviewer report: Reviewer response for version 1

**DOI:** 10.5256/f1000research.159080.r309554

**Published:** 2024-08-24

**Authors:** Sanjiv Luther

**Affiliations:** 1 Department of Immunobiology, University of Lausanne, Lausanne, Switzerland

Piquet and coauthors address in this study the interesting question of how the lymph node (LN) stroma changes or is reprorammed upon draining a tumor site with alterations presumably contributing to tumor metastasis to this site along with immune suppression. They present insightful scRNAseq datasets describing how different murine LN stromal cell types react to either a live or a fixed inoculation with the MC38 colorectal cancer cell line, when compared to a nondraining LN. While both types of inoculation lead to a comparable LN swelling, including a strong amplification of the LN fibroblasts, lymphatic endothelium (LEC) and blood endothelium (BEC), consistent with a strong inflammatory response in both settings, the impact of live tumor cell inoculation shifted much more the proportions and transcriptional profile of distinct subsets of stromal cells compared to injection with fixed tumor cells, pointing to tumor-specific factors driving these changes. The transcriptional shifts were most pronounced among two fibroblast subsets, termed MRC and BRC, as well as among two LEC subsets that are also those thought to be most exposed to the incoming lymph and its factors. The resource provided by the authors should be of great interest to all scientists interested in tumor metastasis or anti-tumor immunity. The manuscript is well written, the data are of high quality and appropriately displayed; and the findings are discussed in an insightful way.

**Major points:**

 1) The clustering of the different subsets of fibroblasts, or lymphatic or blood endothelial cells is presented without any tables or heatmaps showing the top differentially expressed genes for each subset, so that one can judge the cells clustered for example as MRC or BRC. Currently one would be forced to redo all the bioinformatic work again, based on the individual data files provided. Such additional data would help considerably the expert reader to assess and interpret the data and the labeling of the clusters, especially as the frequencies of the FDC and MRC populations look higher compared to other scRNAseq data sets (eg. Rodda/Cyster Immunity 2018) or relative to the analysis by flow cytometry (eg. Huang/Luther, PNAS 2018), also relative to TRC and MedRC. Any histological stainings (with antibodies or by ISH) validating the key scRNAseq data, including the subset identification/clustering would greatly enhance the value of the data; eg. are the MRC-like cells really still restricted to the SCS-lining area, as are the fLEC-phenotype cells

  2) Similarly, no tables are provided as supplement to list the DE genes in the various settings as only part of them are mentioned in the figure 2, and they are limited to the ones showing an increase with none labeled showing a decrease in a live tumor cell draining LN (vs the nondraining LN). This reviewer can imagine that labeling also part of the downregulated genes may render the data and its discussion more complex but then at least tables containing these data should be provided to allow an interested reader to look into them without need to reanalyze all the data. They may contain data relevant for the main question of the paper; eg. how does the LN suppress anti-tumor immunity or get prepared for tumor metastasis. Please improve also the description in the legend so that one understands why the log2 fold changes is negative for transcripts enriched in drLN.

 3) The draining LN response is investigated on day 12 after tumor cell inoculation, comparing live tumor cell vs fixed tumor cell injection which is an elegant approach. As the same number of cells is injected in the setting of live cells as of fixed cells, but the final number of live tumor cells is not stated for d12 (but is presumably much higher, by a factor of several fold), the authors should probably acknowledge in the discussion the large difference in final tumor cell material in the two settings that is likely to contribute to the transcriptional differences observed, besides larger differences in the tissue cells of the primary injection site during the time period upto d12. Thus, the difference in factors infusing the LN is not only due to the live tumor cells but also due to the difference in final tumor cell numbers and their differential effects on the peripheral tissue in the two settings.

 4) It is unclear whether the authors verified if any of the live tumor injected mice showed tumor cell metastasis to the draining LNs which got analyzed. That could have been analyzed by flow cytometry (CD45- cells with specific FSC/SSC characteristics; or a non-stated epithelial marker) or by other means. This information would be valuable to know given the likely difference between premetastatic vs metastatic LNs stroma.

**Minor points:**

1. The results start with a rather lengthy intro and part of it could be incorporated into the introduction where some of these points are already raised.
2. BRC are claimed to populate the interfollicular space; please explain the rational or the publication stating this.
3. Enzymatic digestion (‘as previously described’): please add reference with a more detailed protocol as this aspect is key for appropriate reproduction
4. Mention at beginning that MC38 are a colorectal carcinoma cell line
5. In the conduit section: conduits are not simply fibers (although they appear that way histologically and contain fibers) but have also basement membranes thereby forming true tubes/channels; thus the term fiber is not ideal. Similar, the term ‘transendothelial channels’ is not appropriate even when talking of the SCS where occasionally such channels traversing the SCS can be observed but the majority of conduits do not seem to traverse the endothelium.
6. ‘Exhibited of’ (please drop ‘of’)
7. IL33 discussion: may add another reference showing role of IL33 for memory T cell response (Marx et al.,2024 [Ref 1])
8. Please spell out LDHA and PKM at first mentioning and possibly the reaction they catalyze to inform the non-specialist reader.
9.  Fig.1B and D: I assume these Umaps show data compiled from all 4 groups of mice (please state clearly in legend); it would be of interest to display the Umaps of each group separately (in a supplementary figure) for readers to understand the stromal cell clustering in a 2D space for the 4 groups.

Is the work clearly and accurately presented and does it cite the current literature?

Partly

If applicable, is the statistical analysis and its interpretation appropriate?

Yes

Are all the source data underlying the results available to ensure full reproducibility?

Yes

Is the study design appropriate and is the work technically sound?

Yes

Are the conclusions drawn adequately supported by the results?

Partly

Are sufficient details of methods and analysis provided to allow replication by others?

Yes

Reviewer Expertise:

lymph nodes, stromal cells, immunity, scRNAseq

I confirm that I have read this submission and believe that I have an appropriate level of expertise to confirm that it is of an acceptable scientific standard, however I have significant reservations, as outlined above.

## References

[1] : The alarmin interleukin-33 promotes the expansion and preserves the stemness of Tcf-1+ CD8+ T cells in chronic viral infection. *Immunity* .2023;56(4) : 10.1016/j.immuni.2023.01.029 813-828.e10 10.1016/j.immuni.2023.01.029 36809763

# Response

**Published:** 2025-03-28

**Authors:** Jonathan Chang

**Affiliations:** Oncology, Novartis, Cambridge, MA, USA

**(Major) Reviewer Comment 1:** The clustering of the different subsets of fibroblasts, or lymphatic or blood endothelial cells is presented without any tables or heatmaps showing the top differentially expressed genes for each subset, so that one can judge the cells clustered for example as MRC or BRC. Currently one would be forced to redo all the bioinformatic work again, based on the individual data files provided. Such additional data would help considerably the expert reader to assess and interpret the data and the labeling of the clusters, especially as the frequencies of the FDC and MRC populations look higher compared to other scRNAseq data sets (eg. Rodda/Cyster Immunity 2018) or relative to the analysis by flow cytometry (eg. Huang/Luther, PNAS 2018), also relative to TRC and MedRC. Any histological stainings (with antibodies or by ISH) validating the key scRNAseq data, including the subset identification/clustering would greatly enhance the value of the data; eg. are the MRC-like cells really still restricted to the SCS-lining area, as are the fLEC-phenotype cells.

**Response: **Cluster identification for our dataset was based on findings from previously published studies that have deeply characterized of stromal subsets (utilizing combined scRNAseq and IHC approaches of dissociated and intact lymph nodes respectively).  We aligned expression patterns of the markers validated in these studies with differential expression patterns of our identified clusters to assign subtypes.  Notable markers used for cluster identification are noted below, and bubble plots for each fibroblastic or endothelial cluster are now included in Supp Fig 1 of the revised manuscript as well as the methods section (these Key identifiers also listed below).  Additionally, DEG lists were included in Supplementary table 1 (for fibroblast subclusters) and Supplementary table 2 (for endothelial subclusters). Lymphatic Endothelial Subsets of the LN were annotated based on characterization work from den Braanker, *et al. *2021 (PMID: 34769408) and Fujimoto, *et al.* 2020 (PMID: 32251437).  Key identifiers for each subset include the following: **cLEC**: *ackr4+, cd36+, edn1+, anxa2+*. **fLEC**: *lyve1+, glycam1+, coch+, madcam1+* **Medullary LEC**: *lyve1+, marco+, il33+, itg2b+* **Cortical LEC**: *mrc1+, ptx3+, reln+*. Fibroblastic Subsets of the LN were annotated based on characterization collective work from multiple studies including Rodda, *et al. *2018 (PMID: 29752062), Pikor *et al.* 2021 (PMID: 32424359) and Beuchler *et al.* 2021 (PMID: 33981032).  Key identifiers for each subset include the following: **TRC**: *ccl19+, ccl21+, grem1+* **BRC**: *cxcl13+, cr2-, madcam1-, tnfsf11-, ch25h+* **FDC**: *cxcl13+, madcam1+, coch+, cr2+, tmem119+, pthlh+* **MRC**: *madcam1+, tnfsf11+, enpp2+* **MedRC**: *cxcl12(hi), inmt+, stc1+, bst1-* **PRC**: *cd34+, fndc1+, pi16+, dpp4+* **Activated SC**: *nr4a1+.     *We appreciate the reviewers’ careful consideration of population frequencies within our dataset relative to the above referenced studies.  While we are unable to conclusively account for these discrepancies, we hypothesize that differences in frequencies may relate to 1) distinct methodology for tissue dissociation, 2) differences in cell enrichment prior to scRNAseq, and 3) differences in which specific peripheral LNs are included in the dataset.  Importantly, we do not make any conclusions or assumptions that are specifically about the baseline abundance of stromal subsets relative to other subsets, but rather all of the comments we make regarding population frequency is centered on changes of subset representation between tumor-draining and corresponding non-draining LNs, and thus these potential variables resulting from sample preparation methodology are internally controlled.  We are unfortunately unable to provide comprehensive IHC to corroborate the spatial representation of these markers in our dataset, but IHC of LN tissues exploring many of the above-mentioned markers has been performed in the referenced publications.  Regarding the specific example of MRC vs BRC, the primary distinction between these clusters was a lack of *madcam1* and *tnfsf11* on the subset we identify as “BRCs” relative to MRCs.  Both clusters expressed *cxcl13* and exhibited low expression of markers typically associated with TRC (including *ccl19*/ *ccl21*).

**(Major) Reviewer Comment 2:**   Similarly, no tables are provided as supplement to list the DE genes in the various settings as only part of them are mentioned in the figure 2, and they are limited to the ones showing an increase with none labeled showing a decrease in a live tumor cell draining LN (vs the nondraining LN). This reviewer can imagine that labeling also part of the downregulated genes may render the data and its discussion more complex but then at least tables containing these data should be provided to allow an interested reader to look into them without need to reanalyze all the data. They may contain data relevant for the main question of the paper; eg. how does the LN suppress anti-tumor immunity or get prepared for tumor metastasis. Please improve also the description in the legend so that one understands why the log2 fold changes is negative for transcripts enriched in drLN.

**Response: **Tables including the complete DEG lists of the indicated subpopulations in tdLN vs ndLN have been included in Supplementary table 3 of the revised manuscript.  Regarding log₂ fold change interpretation: In volcano plots (B, D, F), negative log₂ fold change values indicate genes that are more highly expressed in draining lymph nodes (dLNs) relative to non-draining lymph nodes (ndLNs). This is because differential expression was calculated using ndLNs as the reference group. As a result, transcripts upregulated in dLNs are represented on the left side of the plot (negative fold change), while genes enriched in ndLNs appear on the right side (positive fold change).

**(Major) Reviewer Comment 3:**  The draining LN response is investigated on day 12 after tumor cell inoculation, comparing live tumor cell vs fixed tumor cell injection which is an elegant approach. As the same number of cells is injected in the setting of live cells as of fixed cells, but the final number of live tumor cells is not stated for d12 (but is presumably much higher, by a factor of several fold), the authors should probably acknowledge in the discussion the large difference in final tumor cell material in the two settings that is likely to contribute to the transcriptional differences observed, besides larger differences in the tissue cells of the primary injection site during the time period upto d12. Thus, the difference in factors infusing the LN is not only due to the live tumor cells but also due to the difference in final tumor cell numbers and their differential effects on the peripheral tissue in the two settings.

**Response: **The reviewer is correct, in that mice inoculated with “live” tumor cell will invariably harbor more tumor cells (and thus more overall tumor antigen) at the time of harvesting the associated LN due to continued tumor growth.  This is unfortunately difficult to control for experimentally.  We have noted this concern as a potential caveat in the revised manuscript under the subheading “Caveats and study limitations”.

**(Major) Reviewer Comment 4:**  It is unclear whether the authors verified if any of the live tumor injected mice showed tumor cell metastasis to the draining LNs which got analyzed. That could have been analyzed by flow cytometry (CD45- cells with specific FSC/SSC characteristics; or a non-stated epithelial marker) or by other means. This information would be valuable to know given the likely difference between premetastatic vs metastatic LNs stroma.

**Response: **Overt metastatic tumor growth was not observed in any live tumor-bearing mice, nor were any clusters identified as cancer cells within the total 50,381 cells sequenced in this dataset.  However, because these samples were enriched for stromal populations, it is likely that cancer cells (if present) would be lost following sample enrichment, particularly if originally present in low numbers.  As the reviewer suggested, we looked further into our (unenriched) flow cytometry data of dissociated TdLN and ndLNs to identify presence of tumor cells.  While no epithelial marker is included in this panel, MC38 cancer cells can be identified as CD45-, PDPN-, CD31-, PD-L1+ (PMID: 28507803).  Indeed we do find small numbers of these cells specifically in the tumor-draining lymph node.  Average cell counts were 114 total cells in TdLN compared to 4 in NdLN (p value = 0.0085).  This analysis has been added to the revised manuscript as supplementary figure 3.  We believe that presence of cancer cells at such low numbers, may reflect either shed/migrating tumor cells or the presence of early micrometastases.  Discussion of this topic is likewise added to the revised manuscript, and the above-mentioned flow cytometry analysis has been included as Supplementary Fig. 3.

**(Minor) Reviewer Comment 1:**  The results start with a rather lengthy intro and part of it could be incorporated into the introduction where some of these points are already raised.

**Response: **Per the reviewer’s suggestion, we have removed the first paragraph of the results.  This paragraph reiterated the role of tissue stroma in tumor progression and was mostly redundant. 

**(Minor) Reviewer Comment 2:**  BRC are claimed to populate the interfollicular space; please explain the rational or the publication stating this.

**Response: **While the exact subsetting criteria and nomenclature varies across different publications to date, the population we refer to as “BRCs” most closely aligns with interfollicular reticular cell populations described as Ccl19lo, Ch25h+, Cxcl13+ in Pikor *et al.* (PMID: 32424359) and Rodda *et al. *(PMID: 29752062).  This population (or a proportion of this population) is considered to reside in the interfollicular space due to IHC and RNAscope data included in those respective publications.  Regarding the nomenclature, this population of reticular cells is considered a subset of “B cell interacting” reticular cells in Pikor *et al.*  Whereas it is referred to as a subset of TRC in Rodda *et al.*  We use the BRC nomenclature due to low Ccl19 and high Cxcl13 expression, suggestive of a B-cell association. 

**(Minor) Reviewer Comment 3:**  Enzymatic digestion (‘as previously described’): please add reference with a more detailed protocol as this aspect is key for appropriate reproduction.

**Response: **Reference added to revised manuscript

**(Minor) Reviewer Comment 4:** Mention at beginning that MC38 are a colorectal carcinoma cell line.

**Response: **mention of MC38 as colorectal carcinoma cells has been added to the revised manuscript.

**(Minor) Reviewer Comment 5:** In the conduit section: conduits are not simply fibers (although they appear that way histologically and contain fibers) but have also basement membranes thereby forming true tubes/channels; thus the term fiber is not ideal. Similar, the term ‘transendothelial channels’ is not appropriate even when talking of the SCS where occasionally such channels traversing the SCS can be observed but the majority of conduits do not seem to traverse the endothelium.

**Response: **We have changed the description of LN conduits as “a branched network of fibers” to state instead “a branched network of fibrous structures”.  While our discussion of conduit networks does not fully address the intricacies of conduit anatomy, we have included relevant references to more in-depth exploration of this topic.  Regarding the statement of transendothelial channels, we discuss the nature of these structures as described in the referenced citation (Ranktakari *et al* 2015).  We do not make any statement speculating whether any conduits themselves traverse the endothelium.  Rather, we discuss the findings of the referenced study which explore the idea of size-restriction as a function of PLVAP fibrils that exist within transendothelial channels.  To clarify, in this publication, “transendothelial channels” and conduits are separate structures.  The former spans the length of sinus-lining endothelial cells, while the latter underlies the endothelium (as such this finding is not reliant on whether any or all conduits actually traverse the endothelium).  This publication suggests that molecules pass from LN sinus into the LN parenchyma through transendothelial channels, and upon traversing the endothelial lining, can subsequently access the underlying conduit network freely.  However, the structure of the transendothelial channel (dictated by PLVAP) is the gating feature for larger molecules.  We reference this finding because any structural disruption to the sinus-lining endothelium could theoretically perturb the above described regulation of molecule sizes that are allowed to pass into the conduit network.  This would be one potential explanation to the loss of size-exclusivity noted by Riedel *et al* within the tdLN.

**(Minor) Reviewer Comment 6:**  ‘Exhibited of’ (please drop ‘of’).

**Response: **Typo has been corrected.

**(Minor) Reviewer Comment 7:** IL33 discussion: may add another reference showing role of IL33 for memory T cell response (Marx et al.,2024 [Ref 1]).

**Response: **This reference has be added.  Thank you for the suggestion

**(Minor) Reviewer Comment 8:** Please spell out LDHA and PKM at first mentioning and possibly the reaction they catalyze to inform the non-specialist reader.

**Response: **Definitions have been added.

**(Minor) Reviewer Comment 9:** Fig.1B and D: I assume these Umaps show data compiled from all 4 groups of mice (please state clearly in legend); it would be of interest to display the Umaps of each group separately (in a supplementary figure) for readers to understand the stromal cell clustering in a 2D space for the 4 groups.

**Response: **Per the reviewer’s suggestion, we have generated a new supplementary (Supplementary figure 2), which exhibits the distribution of cells from each treatment group within the corresponding endothelial (Supplementary Fig 2A) and fibroblast (Supplementary Fig 2B) Umaps.  Visualization of cell clustering in this 2D space further reflects the increased representation of specific stromal subclusters (including fLEC, MRC, and PRC) from the tumor-draining LN.
